# Effect of Removing Chocolate Milk on Milk and Nutrient Intake Among Urban Secondary School Students

**DOI:** 10.5888/pcd17.200033

**Published:** 2020-08-27

**Authors:** Hannah R. Thompson, Lorrene Ritchie, Esther Park, Kristine A. Madsen, Wendi Gosliner

**Affiliations:** 1 University of California, Berkeley, School of Public Health; 2 Nutrition Policy Institute, Berkeley, California

## Abstract

**Introduction:**

Schools across the United States have removed sweetened, flavored milk from cafeterias to reduce students’ sugar consumption and improve their health. However, evidence on the impact of the removal is limited. We examined the effect of a policy that removed chocolate milk from secondary schools on students’ milk consumption and estimated milk-related nutrient intake.

**Methods:**

We collected data on milk selection and consumption during 1 lunch period in 24 California public secondary schools pre-policy (N = 3,158 students in 2016) and post-policy (N = 2,966 students in 2018). Schools had a student population that was 38% Asian and 29% Latino, with 63% qualifying for free or reduced-price meals. We used linear mixed effects models to assess changes in milk selection and waste, and we estimated related changes in added sugars, calcium, protein, and vitamin D consumed from milk.

**Results:**

The proportion of students selecting milk declined 13.6%, from 89.5% pre-policy to 75.9% post-policy (95% CI for difference, 10.8% tο 16.4%), but the proportion of milk wasted remained stable (37.1% vs 39.3%; 95% CI for difference, −0.2% to 4.6%). Although average per-student milk consumption declined by less than 1 ounce per student (from 4.8 oz to 3.8 oz; 95% CI for difference, −1.1 oz to −0.7 oz), we observed no significant reductions in average per-student intake of calcium, protein, or vitamin D from milk. Estimated added sugars from milk declined significantly, by 3.1 grams per student (95% CI, −3.2 g to −2.9 g).

**Conclusion:**

Removing chocolate milk modestly reduced student milk consumption without compromising average intake of key milk-related nutrients, and consumption of added sugars from milk declined significantly. Secondary schools should consider removing chocolate milk to support healthy beverage consumption.

**Summary**

**What is already known about this subject?**

Removing sweetened, flavored milk from school cafeterias is a popular but somewhat controversial intervention to reduce students’ consumption of added sugars. However, limited rigorous evidence on the practice exists.

**What is added by this report?**

We examined the effect of removing chocolate milk from middle and high school cafeterias in a diverse, low-income school district.

**What are the implications for public health practice?**

Although average milk consumption declined slightly across the student population, related calcium, protein, and vitamin D consumption remained stable, and consumption of added sugars significantly decreased. Removing chocolate milk from secondary school cafeterias is an easily scalable and potentially low-cost intervention to support healthier student beverage consumption.

## Introduction

Sugar-sweetened beverages (SSBs) are one of the leading sources of added sugar in youth’s diets (1), and consumption is causally linked to the development of obesity, cardiovascular disease, type 2 diabetes, and dental caries (2–4). Sixty-five percent of adolescents aged 12 to 19 years reported consuming at least one SSB on a given day (5). Furthermore, racial/ethnic and income-related disparities in SSB intake and obesity persist (5–7). Leading public health and medical organizations, including the World Health Organization, American Heart Association (AHA), and American Academy of Pediatrics, recommend a drastic reduction in SSB consumption to improve health outcomes and reduce obesity (8,9).

Students in the United States consume nearly half of their calories from school meals; thus, schools offer an opportune setting for improving their diets (10). Interventions that target the school food environment, rather than focusing on individual dietary behaviors, have been found most effective at positively affecting dietary intake (11). Sweetened, flavored milks remain the only SSBs available in schools as part of federally reimbursable school meal programs (12). As such, policies to remove chocolate milk in schools are one environmental approach increasingly used to reduce youth SSB consumption. Although chocolate milk has the same key nutrients as plain, unflavored milk, it has up to twice as much sugar.

Some stakeholders worry that eliminating chocolate milk from schools will decrease students’ consumption of essential nutrients, such as calcium and vitamin D, and increase milk waste (13–16). Findings from a few studies validate these concerns (15,17). For example, one study in 11 Oregon elementary schools found that total milk sales decreased and milk waste increased after a chocolate milk removal policy was implemented (18), and research in 4 elementary and middle schools in an urban Massachusetts district found a significant increase in milk waste immediately after chocolate milk was removed from cafeterias (19).

Other studies showed positive effects of chocolate milk removal policies (20,21). Research in a single rural Oregon elementary school reported a significant decrease in consumption of added sugars and only a negligible decrease in calcium and protein intake following chocolate milk removal (21). However, findings from a single elementary school may not be generalizable to larger, urban school districts. Little is known about the effect of removing chocolate milk from middle and high schools, but understanding this effect is important, because teenagers consume more SSBs than younger children (22). Moreover, research is also lacking among low-income students of color, who typically consume more SSBs than their high-income white peers.

During the 2017–18 school year, a large, diverse, urban California school district introduced a policy to remove chocolate milk from all middle and high schools as part of a broad district-wide strategy to reduce students’ intake of added sugar. The purpose of our study was to assess the effect of the school district’s chocolate milk removal policy on student milk selection, waste, and overall consumption and to estimate attendant changes in calcium, protein, vitamin D, and added sugar intake among racially/ethnically and socioeconomically diverse secondary school students.

## Methods

### Setting and participants

We collected data on milk selection and waste as a part of the Multi-Pronged Intervention to Increase Secondary Student Participation in School Lunch (MPI) study (23). All participating schools (n = 24) were traditional middle schools (grades 6–8, n = 12) and high schools (grades 9–12, n = 12) in the San Francisco Unified School District, an urban district in Northern California. The MPI study employed a quasi-randomized design in which 6 middle and 6 high schools received a 3-pronged intervention (lunchroom redesign, additional points of lunch sale [mobile cart and vending machine], and teacher outreach) over a 3-year study period (school years 2015–16 through 2017–18) and 12 schools served as comparisons and did not receive the intervention. We found no evidence of effect modification by MPI intervention school versus comparison school status (*P* > .20); therefore, data for all 24 schools are presented together. This research was approved by the University of California, Berkeley, Committee for the Protection of Human Subjects and the Research, Planning, and Accountability Department of the San Francisco Unified School District.

During the 2017–18 school year, independent of the MPI study, the school district implemented a policy that removed chocolate milk from schools. This policy, part of a broader district initiative to reduce students’ sugar intake (including removing fruit juice from schools and cutting down on the amount of added sugar in foods served as part of school meals), began in middle schools in August 2017 and in high schools in January 2018. High school implementation was delayed by one semester after surveys and interviews with high school students suggested that additional education and engagement would lead to a smoother transition. Pre-policy, schools in the district offered students the choice of either low-fat (1%) plain, unflavored milk or nonfat chocolate milk. Post-policy, students could select either low-fat (1%) or nonfat plain, unflavored milk. All milk came in 8-ounce cartons (weighing 257 grams when full and 11 grams when empty). Each 8-ounce serving of milk (regardless of flavor or fat content) contained 300 milligrams of calcium, 9 grams of protein and 3.1 micrograms of vitamin D; each serving of nonfat chocolate milk contained 11.3 grams of added sugar.

### Data collection

We collected data during 1 lunch period per school in the spring (February–April) of 2016 and 2018. All students participating in the school lunch program were eligible to participate. At the beginning of each lunch period, a maximum of 220 numbered trays were distributed to students waiting in lines for school lunch (students could consent or decline participation at this time). A maximum of 220 trays per school was based on the prior school year’s average school lunch participation to ensure that most or all students could participate in the study if they chose to. Students were selected as they lined up at the beginning of the lunch period until no more students were available to participate. Once students selected their lunch, a researcher recorded the tray number, whether the student selected milk, and the type of milk selected. Students were instructed to return the tray with leftover food, milk, and trash to the research team. When trays were returned, another researcher recorded the corresponding tray number on milk cartons; cartons were weighed to the nearest gram on an OXO Good Grips 11-pound–capacity food scale, and carton numbers and weights were recorded. For each carton of milk returned, the grams of milk wasted were calculated as the full carton weight minus weight when returned, and grams of milk consumed were estimated as 246 grams (calculated weight of milk) minus grams of milk wasted. The proportion of milk wasted was calculated as grams wasted divided by 246 grams.

### Statistical analysis

We used linear mixed effects models to assess differences pre-policy (2016) and post-policy (2018) in 1) the proportion of students who selected milk (the number of students who selected milk divided by the total number of students participating in the study), 2) the proportion of milk wasted (the mean of proportion of milk wasted per carton), and 3) average ounces of milk consumed per student (sum of total grams of milk consumed divided by number of participating students). Additional models estimated the average amounts of calcium, protein, vitamin D, and added sugar consumed from milk per student across the study population. Estimates were based on estimates of the mean number of ounces of milk consumed per student. Additional models examined interaction by school type (middle vs high) and MPI intervention status (intervention vs control school) for each outcome. All models included a random effect for school and were controlled for school-level demographic characteristics (downloaded from the California Department of Education (24), including school type (middle vs high), total school enrollment, student enrollment by race/ethnicity, and proportion of students who qualified for free or reduced-price meals. We used Stata/SE 15.1 (StataCorp LLC) to perform analyses.

## Results

Students in participating schools were diverse (38% of students were Asian and 29% Latino) and predominantly low-income (63% of students qualified for free or reduced-price meals) (Table 1). Pre-policy, 3,158 students participated in lunch data collection across all 24 schools (mean, 132 per school). Post-policy, 2,966 students participated in lunch data collection (mean, 124 per school). Before policy implementation, 90% of participating students selected milk (low-fat, plain, unflavored milk, 48%; nonfat chocolate milk, 52%) (Table 2). After policy implementation, 76% selected milk (nonfat milk, 10%; low-fat, plain, unflavored milk, 83%; cartons missing milk type, 7%).

**Table 1:** Demographic Characteristics, Students in 24 California Public Secondary Schools, Before Chocolate Milk Removal Policy (N = 3,158) and Post-Policy (N = 2,966), the Multi-Pronged Intervention to Increase Secondary Student Participation in School Lunch Study

Characteristic^a^	Baseline, 2015–2016 (Pre-Policy)	Follow-up, 2017–2018 (Post-Policy)	*P* Value^b^ for Difference Between Baseline and Follow-up
**Total student enrollment, no. (SD)**	978 (620)	995 (607)	.92
**Race/ethnicity^c^**			
African American	9.1 (5.1)	7.7 (4.4)	.33
Asian	38.0 (21.0)	36.8 (21.6)	.84
Latino	29.3 (18.7)	30.4 (19.1)	.84
White	10.8 (8.6)	11.5 (9.0)	.78
**Qualified for free or reduced-priced meals**	63.0 (15.1)	57.9 (13.8)	.22
**Average daily school lunch participation**	26.7 (10.9)	25.1 (10.2)	.56

a Values are percentage (standard deviation) unless otherwise indicated.

b *P* value calculated from *t* tests.

c The proportion of students who do not identify as African American, Asian, Latino, or white are not included; therefore, percentages do not total 100.

**Table 2:** Adjusted^a^ Differences in Proportion Selecting Milk, Proportion of Milk Consumed, and Nutrients Consumed From Milk, Students in 24 California Public Secondary Schools, Before Chocolate Milk Removal Policy (N = 3,158) and Post-Policy (N = 2,966), the Multi-Pronged Intervention to Increase Secondary Student Participation in School Lunch Study

Variable	Pre-policy, 2015–2016, N = 3,158	Post-policy, 2017–2018, N = 2,966	Difference (95% CI)
Students participating in the study per school, average number (range)	132 (27 to 214)	124 (58 to194)	−8 (−16.9 to 8.9)
Students who selected milk, % (SD)	89.5 (9.8)	75.9 (9.8)	−13.6 (−16.4 to −10.8)^b^
Proportion of milk wasted, % (SD)	37.1 (10.4)	39.3 (10.7)	2.2 (−0.2 to 4.6)
Milk consumed per student, mean (SD), oz	4.8 (0.7)	3.8 (0.7)	−0.9 (−1.1 to −0.7)
Calcium consumed per student, mean (SD), g	188.7 (25.0)	182.2 (24.7)	−6.5 (−13.7 to 0.7)
Protein consumed per student, mean (SD), g	5.7 (0.7)	5.5 (0.7)	−0.2 (−0.4 to 0.02)
Vitamin D consumed per student, mean (SD), mcg	1.95 (0.3)	1.88 (0.3)	−0.1 (−0.14 to 0.01)
Added sugars consumed per student, mean (SD), g	3.1 (0.5)	0 (0)	3.1 (−3.2 to −2.9)^b^

a Data calculated from linear mixed effects models with a random effect for school, adjusted for secondary school type (middle or high, total school enrollment, student enrollment by race/ethnicity, and proportion of students eligible for free or reduced-price meals).

b *P* < .05.

Results from adjusted models showed that significantly fewer students selected milk after the chocolate milk removal policy was implemented: the proportion of students selecting milk dropped 13.6% (from 89.5% pre-policy to 75.9% post-policy; 95% CI for difference, −16.4% to −10.8%) (Table 2). However, the proportion of milk wasted did not change significantly (37.1% vs 39.3%; 95% CI for difference, −0.2% to 4.6%).

Overall milk consumption declined by 0.9 ounces per student (4.8 oz vs 3.8 oz; 95% CI for difference, −1.1 oz to −0.7 oz). In addition, small but nonsignificant decreases in the average amount of nutrients consumed from milk per student participating in the study were observed: calcium (from 189 g to 182 g; 95% CI for change, −13.7 g to 0.7 g); protein (from 5.7 g to 5.5 g; 95% CI, for change −0.4 g to 0.02 g); and vitamin D (from 1.95 mcg to 1.88 mcg; 95% CI for change, −0.14 mcg to 0.01 mcg). Added sugar consumption from milk significantly declined; after policy implementation, students consumed an average of 3.1 fewer grams of added sugars from milk (95% CI, −3.2 g to −2.9 g).

We saw no evidence of effect modification by school type (middle vs high) for either the proportion of students who selected milk (*P* = .11) or the proportion of milk consumed (*P* = .76).

## Discussion

Ours was the first study to our knowledge to examine the effect of a district-level chocolate milk removal policy on milk selection and consumption among racially/ethnically and socioeconomically diverse middle and high school students. During the year when chocolate milk was removed from school meals, we observed a 14% decline in the proportion of students selecting milk with school lunch. However, among the nearly 75% of students who did select milk at follow-up, we saw no increase in milk waste post-policy. Despite a slight (<1 oz) decrease in student milk consumption across the population after the policy was passed, student intake of milk’s key nutrients (calcium, protein, and vitamin D) at the population level was not reduced. Furthermore, students’ consumption of added sugar from milk declined significantly. These findings suggest that policies removing chocolate milk from school meals could improve dietary intake and health among urban secondary school students.

Although the decline in overall milk consumption we saw in our study was small, it may, nonetheless, overstate any negative effects of removing chocolate milk. Follow-up data from our study were collected during the same year as implementation of the chocolate milk removal policy, which provided only short-term results of changes associated with the policy. Other studies have similarly demonstrated a decrease in milk purchasing during the same year chocolate milk was removed from school meals (18,25). However, in a study conducted in 2 kindergarten-to-8^th^-grade New England schools educating primarily low-income students (20), researchers saw student milk selection increase from 52% immediately after a policy removing flavored milk was implemented to 72% 2 years post-policy (they did not have data on milk selection pre-policy). This suggests that over time, students’ acceptance of such policies may increase, and exclusive availability of plain, unflavored milk in schools may be normalized, leading to a rebound of milk selection and consumption. Repeating measures in our study schools after the policy becomes more established would provide a better understanding of the long-term effect of chocolate milk removal from these secondary school cafeterias.

Although chocolate milk constituted 52% of the milk selected pre-policy, in our study nearly 75% of students continued to select milk after chocolate milk was no longer available. Additionally, overall milk consumption declined by less than 1 ounce per student, and milk waste did not increase, suggesting that offering milk with added sugar is not necessary to promote healthy beverage intake in secondary schools. This is important both for students’ health and also for reducing school food waste and its related negative environmental impacts.

It is important to note that the school district made a substantial effort to ensure buy-in for the policy before implementation, including a phased roll-out that was sensitive to schools’ and students’ needs (ie, delaying implementation in high schools until the policy could be clearly explained and justified to staff and students). Although few studies have described the effect removing chocolate milk had on food waste, Hanks et al (18) reported an increase in milk waste after chocolate milk was removed from 11 Oregon elementary schools. Thus, anticipating opposition to such policies and providing clear and open communication may protect against milk waste and reduced milk consumption.

Furthermore, and not surprisingly, consumption of added sugar from milk significantly declined once chocolate milk was removed from school lunch. Davis et al (21) similarly saw average added sugar intake from milk decrease after chocolate milk was removed from school lunches, with negligible average reductions in calcium consumption. Hanks et al (18) also demonstrated a reduction in students’ consumption of added sugar after chocolate milk was removed from 11 Oregon elementary schools.

Avoiding SSBs is an important strategy to prevent childhood obesity (26). AHA recommends that youth consume only 6 teaspoons (25 grams) of added sugar a day (27). In our study, we estimated a 3.1 gram reduction in added sugar per student per day after the policy, which represents more than 12% of the AHA’s recommended 25-gram limit of added sugar. If students drink a full 8-ounce carton of chocolate milk, they will consume more than a third of the recommended daily limit of added sugar in 1 sitting. Furthermore, calories consumed in liquid form have been shown to have weak satiety properties and stimulate poor energy compensation compared with calories consumed from solid foods (28). Given that adolescents’ SSB consumption is typically higher than recommended, particularly among low-income youth and youth of color (22), removing chocolate milk from school meals could decrease added sugar and SSB consumption, with potentially important implications for public health equity.

Our study had several limitations. First, findings from our sample of 24 secondary schools in 1 urban school district may not be generalizable to rural or less socioeconomically or racially/ethnically diverse districts. Additionally, all of these schools were in a district working to reduce students’ sugar consumption, and half of them also were participating in an intervention that changed cafeteria environments and worked to increase students’ participation in school meals. However, no significant differences in outcomes were observed between schools originally assigned to intervention or control conditions. Second, although data collection days were carefully selected in collaboration with the school district (and menus at each school were aligned to be as similar at baseline and follow-up as possible), factors such as what foods were served for lunch on each data collection day could confound these findings. Furthermore, students did not choose milk in isolation, but rather as a component of a full meal, and we did not assess how this policy may have influenced students’ overall nutrient intake from school lunch. We were also unable to assess whether students compensated by consuming SSBs brought from home or purchased outside of school (42% of study schools — 10 high schools — had open campus policies), though our prior work showed that after SSBs were removed from low-income middle and high schools in California, students did not compensate by drinking SSBs outside of school (29). Our data collection was limited to lunch periods; analogous data were not collected at school breakfasts. Additionally, our study included only schools implementing the chocolate milk removal policy and lacked comparison schools. We do not have data on the number or characteristics of students who declined to participate in the study, which affects the generalizability of these findings. Moreover, with only 24 schools, our study was not adequately powered to examine differences by school-level characteristics. Finally, a lack of data on individual student characteristics precluded a closer examination of student-level factors that could affect milk selection and consumption.

Immediately after chocolate milk was removed from school cafeterias in our study, fewer urban middle and high school students selected milk during school lunch, though milk waste did not increase. Although average milk consumption declined slightly across the population, consumption of calcium, protein, and vitamin D from milk remained stable, while consumption of added sugar from milk decreased significantly. Our findings suggest that secondary schools should consider removing chocolate milk from school lunch to support students in consuming healthier beverages.
